# Factors Associated With Depressive Episode Recurrences in Primary Care: A Retrospective, Descriptive Study

**DOI:** 10.3389/fpsyg.2020.01230

**Published:** 2020-06-05

**Authors:** Shysset Nuggerud-Galeas, Bárbara Oliván Blázquez, María Cruz Perez Yus, Begoña Valle-Salazar, Alejandra Aguilar-Latorre, Rosa Magallón Botaya

**Affiliations:** ^1^Institute for Health Research Aragón (IIS Aragón), Zaragoza, Spain; ^2^Department of Medicine, Psychiatry and Dermatology, University of Zaragoza, Zaragoza, Spain; ^3^Department of Psychology and Sociology, University of Zaragoza, Zaragoza, Spain; ^4^Primary Health Care, Aragón Health Service, Zaragoza, Spain

**Keywords:** depression, primary care, recurrence, influencing factors, retrospective

## Abstract

**Introduction and Objective:**

The early identification of depressive patients having a poor evolution, with frequent relapses and/or recurrences, is one of the priority challenges in this study of high prevalence mental disorders, and specifically in depression. So, this study aims to analyze the factors that may be associated with an increased risk of recurrence of major depression episodes in patients treated in primary care.

**Methods:**

A retrospective, descriptive study of cases-controls was proposed. The cases consisted of patients who had been diagnosed with major depression and who had presented recurrences (*n* = 101), in comparison with patients who had experienced a single major depression episode with no recurrence (*n* = 99). The variables of the study are age at first episode; number of episodes; perception of severity of the depression episode suffered prior to recurrence; number of residual symptoms; physical and psychiatric comorbidity; history of anxiety disorders; family psychiatric history; high incidence of stressful life events (SLEs); and experiences of physical, psychological, or sexual abuse in childhood. The differences of the variables were compared between the case subjects and the control subjects, using the Mann–Whitney, chi-square, and Fisher’s U statistics. A multivariate analysis (ordinary logistic regression) was performed.

**Results:**

The average age of those suffering more than one depressive episode is significantly older (5 years), and a higher percentage of subjects who have experienced more than one depressive episode have a history of anxiety disorders. In the multivariate analysis, the variables that obtained a significant value in the logistic regression analysis were age (OR: 1.03; value: 0.007) and having suffered sexual abuse during childhood (OR: 1.64; value: 0.072).

**Conclusion:**

These indicators should be considered by primary care physicians when attending patients suffering from major depression.

## Introduction

Depression is the most prevalent psychiatric disorder worldwide and is the most common mental illness appearing in primary care (Roca et al., 2009; Kessler et al., 2011). According to the World Health Organization (WHO), approximately 350 million people suffer from depression worldwide (Conejo-Cerón et al., 2017). As for global morbidity, measured in disability-adjusted life years, depressive episodes increased by 37% between 1990 and 2010, and it has been projected that by 2030, it will be the leading cause of disability in developed countries (Conejo-Cerón et al., 2017).

Approximately 20% of all depressed patients develop a chronic course (Kessler et al., 2003). Untreated depression is associated with a decreased quality of life (Gao et al., 2019; Tang et al., 2019; Knight et al., 2020), increased suicide risk (four times more than the general population), and progressively worse health when associated with chronic physical illnesses, etc (Moussavi et al., 2007; Daly et al., 2010). Comorbidity with other chronic conditions (diabetes, hypertension, cardiovascular diseases, and cancer, among others) is high (Cassano and Fava, 2002; Katon, 2003; O’Neil et al., 2015; Read et al., 2017; Zhang et al., 2018) as is the case with other psychiatric diseases such as anxiety disorders (40 to 66%) (Aragonès et al., 2004). There is also a higher incidence of personality disorders in depressive patients than in other populations (Kool et al., 2000). This relationship is mediated by perceived stress (Kim et al., 2016; Thimm et al., 2018) which increases when activities and social support decrease (Chao, 2014; Mayer et al., 2018). In addition to social dysfunction, the majority of people suffering from depression also experience sleep problems (Meerlo et al., 2015; Zhai et al., 2015), weight dysregulation (Ibrahim et al., 2016), and low self-esteem (Sarubin et al., 2018).

Furthermore, diagnosis is complicated, since it is often based on criteria as opposed to very specific symptoms, which may be difficult to recognize by poorly trained professionals. Furthermore, oftentimes, patients do not seek out health assistance due to social stigma (Gabilondo et al., 2011).

It is commonly believed that the probability of experiencing a depressive episode increases with certain genetic, cognitive, medical, environmental, and social factors. These factors may include family history, marital status, gender (being female), postpartum depression, stressful life events, poor social support, chronic organic or mental illnesses, dementia, substance abuse, low level of economic resources, or adverse employment circumstances, among others (Haro et al., 2006; Gili et al., 2013; Melchior et al., 2013). These factors have also been related to an increased probability of recurrence (Bukh et al., 2016a). In addition to these elements, certain other determinants related to recurrence have been found in patients who were previously diagnosed with major depression. These elements are past history of recurrence (Grilo et al., 2010; ten Doesschate et al., 2010; Colman et al., 2011; Karsten et al., 2011; Hardeveld et al., 2013b; van Loo et al., 2015) residual or subsyndromal depressive symptoms (insomnia, sadness, difficulty concentrating, etc.) (Nierenberg et al., 2010; ten Doesschate et al., 2010; Karsten et al., 2011; McClintock et al., 2011; Kiosses and Alexopoulos, 2013; van Loo et al., 2015), child abuse, intensity of previous major depressive episodes, early age of onset (Nanni et al., 2012; Hardeveld et al., 2013a; Bukh et al., 2016b), comorbidities with personality disorders (Grilo et al., 2010; Klein et al., 2013; Bukh et al., 2016a) such as neurotic traits, which have a higher incidence of cases and relapses, mainly in the face of adverse events (Klein et al., 2013), poor psychosocial functioning (Hardeveld et al., 2013a; Stegenga et al., 2013), a feeling of impotence (Colman et al., 2011), and a high incidence of stressful life events or repeated episodic stress (Abravanel and Sinha, 2015; van Loo et al., 2015). The previously described elements indicate that this is a complex and multifactorial disease, and therefore, it has been impossible to determine the different interactions existing between the known risk factors, or the weight of each, based on the distinct circumstances of each subject (Bellón et al., 2008).

Today, most patients suffering from a depressive episode will remit by approximately 50% after 1 year and by approximately 75% after 2 years. While these figures are encouraging, individuals who previously experienced a major depression episode may have high recurrence rates, defined as the appearance of a new major depression episode, following a past episode that had completely remitted. It is important to differentiate between recurrence and relapse, which is defined as the return of the disease when the patient is in the recovery phase. The average recurrence time for the first episode is approximately 3 years and that of the subsequent episode is between 1 and 1.5 years. The risk of recurrence is greater during the first months of recovery (Solomon, 2000). Subsequently, the average recurrence time decreases progressively as the duration of recovery increases. In another study conducted on the Dutch general population, patients who had recovered from a major depression episode were found to have a cumulative recurrence range of 13% in 5 years, 23% in 10 years, and 42% in 20 years (Eaton et al., 2008).

The early identification of depressive patients having a poor evolution, with frequent relapses and/or recurrences, is one of the priority challenges in this study of high prevalence mental disorders, specifically depression (Roca et al., 2011). In addition, primary health care services are the ideal setting for practicing disease prevention strategies (Hardeveld et al., 2013a), including major depression, although there is limited evidence in this area (Gili et al., 2015). Once an initial depression episode has ended, screening mechanisms should be considered in order to quickly identify the factors that may lead to recurrence, in order to reduce morbidity and mortality (Conejo-Cerón et al., 2017). Although this is a relevant mental health problem, few studies are available regarding primary care (Vuorilehto et al., 2005, 2009; Conradi et al., 2008; Gili et al., 2011; Roca et al., 2011) and those focusing on recurrences predominate (Simon, 2000; Gopinath et al., 2007). So, this study aims to analyze the factors that may be associated with an increased risk of recurrence of major depression episodes in patients treated in primary care.

## Materials and Methods

### Design

A retrospective, descriptive study was proposed. Patients who were previously diagnosed with major depression and who had presented recurrences, and patients who had experienced only one major depression episode with no recurrence have been compared.

### Population, Sample, and Sample Size

This study was conducted in a health center located in northern Spain, which was accessed by a population of approximately 26,000 individuals, including 18,775 adults, during 2018. The inclusion criteria were: (1) Over the age of 18, (2) receiving at least one major depression diagnosis, based on the DSM-V criteria, at some point in their life (past or present), and (3) having signed the informed consent.

The diagnosis of depression had to be elaborated by the family doctor. Therefore, recurrence has been considered any new depression episode (after having suffered an initial episode) in which remission has occurred, with said remission being sufficiently long so as to assume that recovery had taken place (Frank et al., 1991; Bockting et al., 2015; Buckman et al., 2018).

The exclusion criteria were: (1) subjects with comorbidity with serious psychiatric diseases (schizophrenia and/or bipolar disorder), (2) dysthymia, and (3) patients who were referred to and are being followed up on psychiatry, in order to establish the focus of “primary care patients.”

The study was carried out at one health center. In 2018, there were 1,100 subjects over the age of 18, who, according to the diagnosis made by their primary care physician, had experienced one or more episodes of depression/depressive disorders (diagnosis P76 according to the International Classification of Primary Care). Therefore, assuming an error of 5% and a 95% probability of success, with a confidence level of 95% and a precision of 3%, and adding 10% for potential mistakes while completing the questionnaires, at least 190 individuals were needed. Finally, 200 subjects participated in the study, exceeding the necessary sample size.

### Variables and Instruments

Socio-demographic variables, current lifestyles, state of depression, and social support data were collected. These variables were considered, since, although not necessarily the same as they were when the subjects suffered from the depressive episode/s, they are related to subjectivity in the perception of health (Asensio-Martínez et al., 2020) and they permit comparison of both groups. In addition to these variables, issues related to the depressive episodes suffered and to recurrence were also collected; this includes clinical data related to the major depression disorder (age at first episode, number of episodes, perception of severity of the depression episode suffered prior to recurrence, and number of residual symptoms). These variables were related to the sole depression episode suffered or to the episode experienced prior to recurrence. If subjects had experienced more than one recurrence episode, the variables were collected based on the episode occurring prior to the most serious recurrent episode. Physical and psychiatric comorbidity; history of anxiety disorders; family psychiatric history; high incidence of stressful life events (SLEs); and experiences of physical, psychological, or sexual abuse during childhood were also analyzed. Table 1 presents the study variables and instruments used; however, each of these variables is explained below.

**TABLE 1:** Study variables and instruments used.

Variables and Instruments	
Socio-demographic variables	Gender Age Country of birth Marital status Coexistence at home Education level Employment status Monthly family income
Variables related to lifestyle	Physical exercise Smoking Alcohol Drugs
Presence of active major depression episode	Hamilton Depression Rating Scale (HDRS)
Social support	Medical Outcomes Study—Social Support Survey (MOS Scale): – Emotional support – Material/instrumental support – Social relations/leisure/distraction – Affective support – MOS Global Index
Clinical variables related to depression	Number of depression episodes suffered Age at onset of first depression episode Subjective perception of severity of the depression episode Number of residual symptoms
Physical and psychiatric comorbidity	Current serious physical or psychiatric illness (personality disorder, obsessive–compulsive disorder or other, to be specified)
History of anxiety disorder	Anxiety crisis or repetitive anxiety states or post-traumatic stress
Family psychiatric history	If the individual had a family history of serious psychiatric pathology
High incidence of stressful life events (SLE)	If the precipitating factor for the episode was an SLE Have experienced SLE events during the past year, using the Social Readjustment Rating, and counting the number of SLEs
Childhood experiences of physical, psychological, or sexual abuse	Childhood Trauma Questionnaire

*Socio-demographic variables*. gender (male, female); age; country of birth; marital status (married or in a relationship, single, separated, widowed); coexistence at home (alone, with a partner, with a partner and children, with relatives, with neighbors or friends, in a residence, others); education level (cannot read or write, no formal studies but can read and write, primary studies, secondary studies, university studies, others); employment status (student, housewife, unemployed with subsidy, unemployed without subsidy, employee, employee with temporary work disability (TWD), disabled, permanent disability, retired, others); type of contract for employed subjects (indefinite contract, temporary contract of more than 6 months, temporary contract of less than 6 months, temporary unspecified, self-employment, other contractual relationships); monthly family income (less than 1000 euros, between 1000 and 2000 euros, more than 2000 euros).

*Variables related to lifestyle*. were also collected. These variables were practicing physical exercise, smoking, alcohol, and drugs. Practicing physical exercise was considered based on the hours of physical exercise (understood as a moderate-intense physical activity) performed on a weekly basis. It was categorized by less than 3 h a week, between 5 and 7 h a week, and more than 7 h a week. Smoking was categorized by the number of cigarettes smoked per day, and subjects were subsequently recorded as smokers or non-smokers. Alcohol consumption was measured in Standard Drinking Units (SDU) consumed weekly, and grams of alcohol consumed per week were subsequently calculated. Individuals were classified into three consumption risk groups (Spanish Ministry of Health Social Services Equality, 2011): low risk consumption (less than 17 SDUs per week for men and 11 for women), dangerous consumption (between 17 and 28 SDUs a week for men and between 11 and 17 SDUs for women), and risky consumption (more than 28 SDUs a week for men and 17 for women). Drug use was coded as non-consumption/sometimes/regularly.

*The non-remission or presence of an active major depression episode*. at the time of data collection was assessed using the Hamilton Depression Rating Scale (HDRS) of 17 items (Hamilton, 1967) validated in Spanish. This scale was found to have good psychometric properties (Ramos-Brieva and Cordero-Villafafila, 1988; Bobes et al., 2003), and it contains 17 items that are assessed from absent (0) to very severe (3), with scores ranging from 0 to 51. The higher the score, the higher the intensity of the depression.

*Social support*. was assessed using the Medical Outcomes Study—Social Support Survey (MOS Scale) developed by Sherbourne and Stewart (1991), using the adapted Spanish language version of Revilla et al. (2005) with a consistency (in Cronbach’s alpha values) of 0.94 for emotional support, 0.85 for affective support, and 0.87 for material support. In our sample, the Cronbach’s alpha for the global score was 0.954, which may be considered very reliable. This scale consists of 20 items, responded to by the subject with never, rarely, sometimes, most of the time, or always, to be subsequently converted to a range of 1 to 5, respectively. The social support score is obtained by adding the results, with the global maximum social support index corresponding to 94 points and minimum social support index corresponding to 19. Emotional support scores are also obtained using this scale, with a maximum of 40 and a minimum of 8 points. Material help has a maximum of 20 and a minimum of 4 points; social leisure and distraction relations can obtain a maximum of 20 and a minimum of 4 points; and emotional support can receive a maximum score of 15 and a minimum of 3 points.

*Clinical variables related to depression*. The following data were collected: Number of depression episodes suffered, as recorded in the computerized medical history; age at the onset of the first depression episode, as recorded in the computerized medical history; subjective perception of severity of the single depression episode for the controls or severity of the episode prior to recurrence, using a Visual Analog Scale of 1 to 10; number of residual symptoms after the sole depression episode or the episode before the recurrence. These symptoms are based on the 17-item Hamilton Depression Rating Scale (HDRS) (Hamilton, 1967), which is designed to quantitatively assess the severity of symptoms and patient changes (Purriños, 2013). Symptoms related to anxiety, somatization, and sleep problems were combined. So, the 14 items that measured residual symptoms were depressed mood, feeling of guilt, suicide, sleep problems, work and activities, retardation, agitation, anxiety psychic, somatization, somatic symptoms gastrointestinal, genital symptoms, hypochondriasis, loss of weight, and insight.

The 14 possible residual symptoms were bad mood, feelings of guilt, suicidal thoughts, sleep problems, decreased work or academic performance, difficulty concentrating or with motor activity, feeling restless, feeling tense or irritable, somatization, decreased appetite, decreased sexual interest, menstrual changes, perceived lability in health, weight loss, being depressed or sick. These symptoms are based on the 17-item Hamilton Depression Rating Scale (HDRS) (Hamilton, 1967) which is designed to quantitatively assess the severity of symptoms and patient changes (Purriños, 2013). Symptoms related to anxiety, somatization, and sleep problems were combined.

*Physical and psychiatric comorbidity*. at the time of data collection: Subjects were asked if they suffered from any current serious physical or psychiatric illness (personality disorder, obsessive–compulsive disorder or other, to be specified). Responses were coded as yes/no.

*History of anxiety disorder*. Subjects were asked if they experienced or had previously experienced an anxiety crisis or repetitive anxiety states or post-traumatic stress. This question was used to detect anxiety disorders. Responses were recorded as yes/no.

*Family psychiatric history*. collected by asking if the individual had a family history of serious psychiatric pathology (specifying schizophrenia, bipolar disorder, personality disorder or other, to be specified). The response was recorded as yes/no.

*High incidence of stressful life events*. Subjects were asked about two aspects: (1) If the precipitating factor for the first or only depression episode was an SLE; and (2) have experienced stressful life events during the past year, as collected from the Social Readjustment Rating Scale by Holmes and Rahe (1967) using the adapted Spanish language version (González De Rivera and Morera, 1983). The Cronbach’s alpha obtained in this study is 0.654, considered to be of moderate reliability. This scale consists of 61 items responding to 61 events, both positive and negative, each of which is weighted based on its severity (from 32 points for the item with the lowest score, corresponding to changes in political opinions and 92 points for the item with the highest score, corresponding to the death of a partner). The higher the score, the greater the suffering resulting from the stressful life events. The number of SLEs experienced was also collected.

*Childhood experiences of physical, psychological, or sexual abuse*. These data were collected using the Childhood Trauma Questionnaire, a retrospective, self-administered questionnaire, validated by Bernstein and Fink (1998), using the Spanish language version (Hernandez et al., 2013). It contains three items that assess past experiences of physical, psychological, and sexual abuse, with the following options: never, rarely, sometimes, often and frequently. Responses were recorded as having experienced or not experienced physical, psychological, or sexual abuse during childhood.

### Procedure

The 1,100 subjects who met the study’s inclusion criteria were contacted by telephone, with 500 of these individuals responding. Of these, 162 refused to participate in the study, and 138 were immobilized or suffered from cognitive impairment that prevented them from completing the survey or had passed away during the previous months. The remaining subjects were contacted by doctors from the health center in order of their medical history number until the necessary sample size was reached. Potential participants were summoned to the health center to administer the data collection notebook. If they were unable to complete it at this time, they were allowed to take it home, and upon completion, it was delivered in a sealed envelope at the health center. Field work took place throughout 2018.

### Statistical Analysis

Statistical analyses were carried out using the IBM^®^ SPSS^®^ Statistics version 22.0.0.0 and Microsoft Excel computer programs. First, the sample distribution was analyzed, obtaining Shapiro–Wilk statistic values that were higher than 0.05 for all of the variables, except number of cigarettes consumed per day for smokers, UBEs consumed weekly, number of depression episodes suffered, and in the MOS scale. Therefore, non-parametric statistics were used. Subsequently, a descriptive analysis was performed: in cases of quantitative variables, mean and standard deviations were used; frequency and percentages were used for qualitative variables. The differences in the variables described above were compared between the two groups, subjects experiencing a single episode and those with more than one episode, using the Mann–Whitney, chi-square, and Fisher’s U statistics. Multivariate analysis was performed using binary logistic regression with forward conditional method. The dependent variable was one or more than one depression episodes (recurrences) and the independent variables were all variables that were statistically significant in the bivariate analysis or that could have a plausible clinical implication. Ultimately, they were age and history of anxiety disorder. Model calibration was carried out using the Hosmer–Lemeshow statistic. The discriminatory power was assessed using the area under the receiver–operator characteristics (ROC) curve obtained by analyzing the probability of the value predicted by the multivariate model. The results of the multivariable model were adjusted by physical, psychological, and/or sexual abuse during childhood (Karsten et al., 2011; Nanni et al., 2012; van Loo et al., 2015). We present an odds ratio and its 95% confidence interval.

### Ethical Aspects

The authors assert that all procedures contributing to this work comply with the ethical standards of the Clinical Research Ethics Committee of Aragón (part of the Department of Health of the Government of Aragon, Spain) and with the Helsinki Declaration of 1975, as revised in 2008. The Study Protocol was approved by the Clinical Research Ethics Committee of Aragón (Spain) (PI18/029). All of the subjects completed an informed consent form, and their data were anonymized.

## Results

A total of 200 individuals participated, of whom, 99 had suffered from a single major depression episode and 101 had experienced more than one depression episode. Of these 200 participants, 147 were women (73.5%) and 35 were men (26.5%). The average sample age was 61.35 years (SD: 15.09, range: 26–88). Table 2 presents the total sample description, based on socio-demographic and lifestyle variables of the patients. The profile of the participant was a woman, born in Spain, whose average age is 61, married, with primary or secondary education, who exercises 1 to 3 h a week and does not smoke, drink alcohol, or consume drugs. The average score on the HDRS is 12.12 (DT: 7.05), indicating a state of mild depression. And the average perceived social support is 74.52, ranging from 19 to 94.

**TABLE 2:** Description of the sample in the socio-demographic, lifestyle, current depression, and social support variables.

Variables	Percentage Mean (SD)
**Gender (%)**
Male Female Age	26.5% 73.5% 61.35 (15.09).
**Marital status (%)**
Single Married or in couple Separated or divorced Widowed	14% 55.5% 17.5% 13%
**Coexistence at home (%)**
Alone With a partner With a partner and/or children With relatives Others	22.5% 33.5% 28.5% 10.5% 5%
**Education level (%)**
Cannot read or write No formal studies but can read and write Primary studies Secondary studies University studies	0.5% 9.5% 55% 22% 13%
**Employment status (%)**
Student Housewife Unemployed with benefits Unemployed without benefits Employee Employee with TWD Retired Permanent disability Others	1% 23% 5% 5.5% 22.5% 5.5% 27.5% 5% 5%
**Type of laboral contract**
Indefinite contract Temporary contract <6 months Temporary contract >6 months Temporary contract unspecified Self-employment Other	68.3% 4.5% 4.5% 3% 13.6 6.1
**Monthly family income**
<1000 euros Between 1000 and 2000 >2000 euros	40.7% 43.7% 15.6%
**Country of birth**
Spain Other country	89.4% 10.6%
**Physical exercise (%)** None
1–3 h/week 5–7 h/week >7 h/week	0% 70% 22% 8%
**Smoker (%)**
No Yes Number of cigarettes per day (mean, SD)	78.5% 21.5% 13.81 (10.37)
**Alcohol consumption (%)**
Low risk Dangerous consumption Risky consumption SDU (mean, SD)	93.5% 4.5% 2% 7.03 (6.50)
**Drug consumption (%)**
Non-consumption Regularly Sometimes	98% 1% 1%
**Active episode of depression** (HDRS) (mean, SD)	12.12 (7.05)
**Perception of social support** (mean, SD)
Emotional support Material/instrumental support Social relations/leisure/distraction Affective support MOS Global Index	31.56 (8.81) 15.41 (4.98) 15.20 (4.76) 12.36 (3.38) 74.52 (20.56)

*SD, standard deviation; HDRS, Hamilton Depression Rating Score; TWD, temporary work disability.*

Table 3 presents the description of the sample with regard to variables related to depression episodes and their recurrences. The average age of onset of the first depression episode is 44.86. Participants experienced between 1 and 4 depression episodes. Of the subjects suffering from more than one depression episode, 52.53% had experienced 2 episodes, 31.31% three episodes, and 16.16% four episodes. The sample tended to perceive the severity of the single episode or the one occurring prior to the recurrence as 6.27, on a scale from 1 to 10. The residual symptoms persisting after the last depressive episode were 5.40 out of 14. Most (73.5%) of the first depression episodes were triggered by an SLE. The average of the SLE experienced over the past year is 3.35 (SD: 3.05), corresponding to an average score of 204.70 (SD: 175.44) points.

**TABLE 3:** Description of the sample for variables related to depression episodes and their recurrences.

Variables	Percentage Mean (SD)
**Severe current disease (%)**
No Yes	70% 30%
**History of anxiety disorder (%)**
No Yes	84% 16%
**Family psychiatric history (%)**
No Yes	68% 32%
**Number of depressive episodes suffered (%)**
1 episode 2 episodes 3 episodes 4 episodes	49.5% 27% 15.5% 8%
**Age at onset of first depressive episode**
(Mean, SD)	44.86 (16.70)
**Perception of severity of first episode**
(Mean, SD)	6.27 (2.37)
**Number of residual symptoms**
(Mean, SD)	5.40 (3.48)
**SLE precipitating first episode (%)**
No Yes	26.5% 73.5%
**Number of stressful events**(Mean, SD) Score on stressful events scale (Mean, SD)	3.35 (3.05) 204.70 (175.44)
**Physical abuse during childhood (%)**
No Yes	74% 26%
**Psychological abuse during childhood (%)**
No Yes	65.5% 34.5%
**Sexual abuse during childhood (%)**
No Yes	92.5% 7.5%

*SD, standard deviation; HDRS, Hamilton Depression Rating Scale.*

Table 4 compares the variables between patients who have experienced a single depressive episode and those who have suffered more than one depressive episode. As shown, there are no differences in the variables analyzed, except for age and history of anxiety. The average age of those experiencing more than one depressive episode is significantly older (5 years) and a higher percentage of subjects who experienced more than one depressive episode have a history of anxiety disorders.

**TABLE 4:** Differentiating characteristics between individuals experiencing one depressive episode and those experiencing more than one.

Socio-demographic Variables	Percentage/Mean (SD)		
	
	Single episode, *N* = 99	More than one episode, *N* = 101	*p*-value
**Gender (%)**
Male Female	27.3% 72.7%	25.7% 74.3	0.806
Age (mean, SD)	58.75 (15.91)	63.91 (13.86)	**0.025**
**Marital status (%)**
Single Married or in couple Separated or divorced Widowed	14.1% 54.5% 17.2% 14.1%	13.9% 56.4% 17.8% 11.9%	0.970
**Coexistence at home (%)**
Alone With a partner With a partner and/or children With relatives Others	22.3% 30.3% 30.3% 13.1% 4%	22.8% 36.6% 26.7% 7.9% 5.9%	0.590
**Education level (%)**
Cannot read or write No formal studies but can read and write Primary studies Secondary studies University studies	1% 10.1% 53.5% 18.2% 17.2%	0% 8.9% 56.4% 25.7% 8.9%	0.278
**Employment status (%)**
Student Housewife Unemployed with benefits Unemployed without benefits Employee Employee with TWD Retired Permanent disability Others	1% 21.2% 4% 7.1% 24.2% 7.1% 24.2% 6.1% 5.1%	1% 24.6% 5.9% 4.0% 20.8% 4% 30.7% 4% 5%	0.870
**Monthly family income (%)**
<1000 Between 1000 and 2000 >2000	41.8% 40.9% 17.3%	39.6% 47.5% 12.9%	0.488
**Physical exercise (%)**
1–3 h/week 5–7 h/week >7 h/week	67.7% 23.2% 9.1%	72.3% 20.8% 6.9%	0.799
**Smoker (%)**
No Yes Number of cigarettes per day (mean, SD)	81.8% 18.2% 14.06 (8.22)	75.2% 24.8% 13.64 (11.84)	0.258 0.611
**Alcohol consumption (%)**
Low risk Dangerous consumption Risky consumption SDU (mean, SD)	58.6% 16.2% 25.3% 6.74 (7.77)	54.5% 21.8% 23.8% 7.31 (5.06)	0.598 0.097
**Drug consumption (%)**
No Yes Sometimes	98% 0% 2%	98% 2% 0%	0.135
**Active depression episode**
(HDRS) (mean, SD)	12.21 (7.55)	12.03 (6.57)	0.842
**Perception of social support**
(mean, SD) Emotional support Material/instrumental support Social relations/leisure/ distraction Affective support MOS Global Index	31.86 (8.80) 15.55 (4.96) 15.24 (4.84) 12.46 (3.26) 75.11 (20.78)	31.27 (8.85) 15.27 (5.02) 15.15 (4.71) 12.26 (3.51) 73.94 (20.43)	0.585 0.678 0.827 0.651 0.515
**Variables related to episodes of depression and recurrences**			
**Severe current disease (%)**
No Yes	67.7% 32.3%	72.3% 27.7%	0,478
**History of anxiety disorder (%)**
No Yes	90.9% 9.1%	77.2% 22.8%	**0.017**
**Family psychiatric history (%)**
No Yes	64.6% 35.4%	71.3% 28.7%	0.314
**Age at onset of first depressive episode**
(mean, SD)	43.48 (17.52)	46.21 (15.83)	0.199
**Number of residual symptoms**
(mean, SD)	5.32 (3.64)	5.48 (3.33)	0.699
**Perception of severity**
(mean, SD)	6.23 (2.39)	6.31 (2.37)	0.615
**Precipitating first episode (%)**
No Yes	26.3% 73.7%	26.7% 73.3%	0.940
**Number of stressful events** (mean, SD) **Score stressful events scale** (mean, SD)	3.38 (2.65) 210.49 (162.99)	3.32 (3.32) 199.03 (187.49)	0.314 0.305
**Physical abuse during childhood (%)**
No Yes	72.73% 28.27%	75.25% 25.75%	0.866
**Psychological abuse during childhood (%)**
No Yes	64.65% 35.35%	66.34% 33.66%	0.984
**Sexual abuse during childhood (%)**
No Yes	94.95% 5.05%	90.10% 9.90%	0.286

*HDRS, Hamilton Depression Rating Scale. Mann–Whitney U statistic for continuous variables and chi-squared and Fisher’s U statistic for qualitative variables. Bold font indicates statistical significance.*

In the multivariate analysis presented in Table 5, the variables that obtained a significant value in the logistic regression analysis were age (OR: 1.03; value: 0.007) and having suffered from sexual abuse during childhood (OR: 1.64; value: 0.072).

**TABLE 5:** Multivariate analysis of the factors associated with recurrences.

**Recurrences**	**Odds ratio**	***p*-value**	**Higher confidence interval**	**Lower confidence interval**
Age	1.03	0.007	1.01	1.05
Sexual abuse during childhood	1.64	0.072	0.96	2.79

## Discussion

This study analyzes the risk factors for recurrence of major depression in primary care. Despite the fact that this is a relevant mental health problem, studies in primary care are limited (Vuorilehto et al., 2005, 2009; Conradi et al., 2008; Gili et al., 2011; Roca et al., 2011) and those focusing on recurrences predominate (Lin et al., 1998; Simon, 2000; Gopinath et al., 2007). In general, the percentage of recurrence in primary care varies depending on the follow-up time and health care level: from 27% in 18 months (Vuorilehto et al., 2009) to 64% at 23 years (Yiend et al., 2009) in primary care; or 25% annually and 85% with 15 years of follow-up in specialized care (Mueller et al., 1999). In the general population, 43.2% of recurrences took place at 2 years (Spijker et al., 2002).

Among the different variables analyzed for subjects suffering from recurrences and those who had not, it has been found that the age and history of anxiety disorders differ significantly between these two groups of patients. Sexual abuse during childhood also explains the recurrence of major depression episodes. As for age, being older is associated with more recurrences, and each successive episode increases the risk for subsequent episodes from an increased risk. Age has been considered a risk factor for the occurrence of depressive episodes by numerous authors (Gum et al., 2009; Kessler et al., 2010; Trainor et al., 2013). On the other hand, a study conducted by Solomon et al. (2000) revealed that 66% of the subjects having an initial depression episode experienced at least one recurrence over the 10 years of follow up. Other studies (Mueller et al., 1999; Eaton et al., 2008; Buckman et al., 2018), however, have suggested a lower prevalence, with recurrences occurring in approximately 33%. Other socio-demographic variables such as gender, marital status, cohabitation, educational level, employment status, and income level have not been found to be relevant in this study on recurrences. These results appear to be in line with those from other studies, which advocate the importance of these factors in the appearance of the first major depression episode but not in its recurrence (Burcusa and Iacono, 2007; Conradi et al., 2008; Vuorilehto et al., 2009; Karsten et al., 2011; Hardeveld et al., 2013a) or relapse (Gopinath et al., 2007; Richards, 2011). Some studies have found differences based on educational level and economic status (Roca et al., 2011; Stegenga et al., 2013).

The second variable found to have predictive power was the history of anxiety disorder. Numerous studies have found this relationship (Lin et al., 1998; Simon, 2000; Stegenga et al., 2013) as well as comorbidity (Burcusa and Iacono, 2007; Gopinath et al., 2007; Conradi et al., 2008). Thus, the appearance of one depression or anxiety episode has been found to predict the occurrence of the other, respectively; and the concurrence of both increases this predictability, questioning the supposed causal relationship of anxiety over depression (Karsten et al., 2011). Our results are consistent with the high comorbidity found between anxiety and depression (Roca et al., 2009; Gabilondo et al., 2010). Some authors have suggested the lack of nosological identity or nosological confusion of both. Furthermore, the masking role of somatic disorders should be considered (Gili et al., 2011).

In the multivariate analysis, in addition to gender, sexual abuse during childhood also had a significant explanatory value for recurrence. Other studies coincide with these results (Kessler and Magee, 1994; Andrews, 1995; Bernet and Stein, 1999; Bifulco et al., 2002). The scientific literature is extensive with regard to the devastating effects of sexual abuse during childhood. These effects include poorer physical and psychological health, including major depression and anxiety disorders; past abuse is also associated with an earlier age of first depression episode and poorer functional status, etc (Springer et al., 2003; Harkness et al., 2006). Sexual abuse is a predictor of successive depression episodes, since these episodes are stressful on their own (Hammen, 1991; Monroe and Harkness, 2005). However, childhood sexual abuse tends to be hidden by patients or goes undetected by their doctors (Springer et al., 2003). If this detection is not improved, it will not serve as a predictor, so whenever early-onset depressive episodes occur, it should always be considered, especially when recurrences take place. The presence of sexual abuse and age in the regression model, but not the other variables examined in this study, may also be considered in light of the hypothetical causal mechanisms of risk factors for recurrence of major depression. The lack of a relationship between stressful life events and the recurrence of depression would be in line with Post’s sensitization hypothesis (Post, 1992).

No significant differences were found between the other clinical and socio-demographic variables examined. Comorbidity with a serious physical or psychiatric illness did not appear to be a relevant factor, in contrast with other studies (Simon, 2000; Gili et al., 2011). This is an area filled with conflicting results, with some finding relationships in the case of dysthymia but not in others such as anxiety or behavioral disorders, the influence of age, or the mediating effect of physical dysfunctionality (Simon, 2000; Burcusa and Iacono, 2007; Hardeveld et al., 2013a). In some studies, psychiatric family history has been found to be relevant, with most of the scientific literature focusing on major depression episodes, especially the early onset of the first episode (Roca et al., 2011; Hardeveld et al., 2013a). In other studies, however, these effects have not been found, and it is necessary to clarify their role and determine if they only refer to certain types of psychiatric morbidity but not to others (Burcusa and Iacono, 2007; Gopinath et al., 2007; Conradi et al., 2008).

Results regarding residual symptoms and the perception of severity of the last episode are equally uneventful. So, although differences have been noted based on the severity of the depression (Conradi et al., 2008; Hardeveld et al., 2009; Vuorilehto et al., 2009; Richards, 2011; Roca et al., 2011), they have only been found with regard to residual symptoms in hospitalized or short-term patient samples (Lin et al., 1998; Burcusa and Iacono, 2007; Hardeveld et al., 2009). On the other hand, the age at the onset of the first major depression episode is a common variable in the few studies available on the prevention of depression recurrence (Lin et al., 1998; Hardeveld et al., 2009; Roca et al., 2011). The findings, however, are divergent, and although there seems to be a tendency to implicate age with recurrences, these recurrences may actually be related to the different designs used or the lack of control over the number of episodes suffered (Lin et al., 1998). Different results may be found, from those in which only the previous number of episodes or the age at onset are predictors, to those in which both show relevance. Our findings, however, are consistent with those of the systematic review and meta-synthesis carried out by Buckman et al. (2018). In this study, strong evidence was found for three factors associated with an increased risk of recurrence in depression: a history of childhood abuse, residual depressive symptoms at the end of treatment, and a history of recurrence. Future studies are needed to clarify the potential mediating role of the number of episodes suffered at the age of onset of the first episode in the recurrence (Burcusa and Iacono, 2007).

In our study, stressful life events were recorded in order to determine those individuals with a high incidence of these events, but their relationship with recurrence could not be objectified. Hardeveld et al. (2009) did not find a significant relationship between this type of events and depression recurrences, but most studies suggest that a relationship exists (Gilman et al., 2003; Burcusa and Iacono, 2007; Roca et al., 2011; Moffett and Mill, 2014). However, there are potential mediating factors that have not been controlled for in our study, such as the type of stressful life event or the age at which it occurred. Low levels of social support have proven to be a risk factor in certain studies (Conradi et al., 2008; Hardeveld et al., 2009). However, results are contradictory and some advocate a common genetic predisposition for both factors, recurrence and social support, or their association only in the case of more severe depression disorders (Burcusa and Iacono, 2007). The lack of a relationship between social support and recurrence would be in line with the lack of a relationship found for the marital status of married or with a partner in this sample.

Our study offers some important strengths: the primary care context, a relevant area in the prevention of recurrence of major depression; the large number of variables collected for patients, including those that are most relevant to recurrences, collected from the various studies of the scientific literature; the use of a standardized diagnostic system; and the exclusion of psychiatric morbidity such as schizophrenia, bipolar disorder or dysthymia, which may potentially distort the results. But the study also has its limitations, mainly, its retrospective style, which may lead to possible memory biases or the impossibility of collecting variables during the period between episodes, according to the chronological moment in which they occurred. A prospective cohort design would be more powerful. However, given that follow-ups should be conducted every 3 to 5 years, which is the average time between depressive episodes (Solomon et al., 2000), this requires large sample sizes and considerable resources. As for stressful events, this information was collected if the first episode was caused by an SLE, and those stressful life events experienced over the past year were also collected in order to determine a high incidence of the same. However, it was not feasible to determine the existence of these events between episodes. Variables related to the existence of depressive episodes such as lifestyle habits (alcohol, tobacco, and drug consumption), current depression, and social support at the time of data collection were not necessarily the same at the time of experiencing the depressive episodes, but this is a means of comparing both groups of patients. Significant differences were not found between them. We attempted to minimize these limitations by collecting information on certain variables from the patient’s medical history. The validity of retrospectively collecting data on social support at the time of the depression episode is quite questionable, so information on social support was collected at the time of the data collection. Therefore, we are unable to determine if social support prior to recurrence is a risk factor, as other studies support (Noteboom et al., 2016).

Finally, it should be emphasized that, although major efforts are being made to identify the risk factors for recurrence of major depression, considerable controversy continues to exist. This disagreement may be due to the great methodological heterogeneity: studies including treatment, different definitions of depression and recurrence, different sources of the sample and age of the subjects and the presence (or lack) of comorbidity. According to Conradi et al. (2008), another potential cause of disagreement is the fact that recurrence is not a unique and homogeneous construct, but rather, various risk factors may be associated with its differential dimensions.

## Conclusion

Our study reveals that age and a family history of anxiety are related to the recurrences of depression. Sexual abuse experienced during childhood may also explain the recurrence of major depression. These indicators should be considered by primary care physicians when caring for patients suffering from major depression. The absence of other risk factors highlights the need for additional longitudinal and prospective studies, which should contain a greater number of variables and extensive follow-up and consider the distinct results on recurrence of major depression.

## Data Availability Statement

The datasets generated for this study are available on request to the corresponding author.

## Ethics Statement

The study was approved by the Ethical Research Committee of Aragon, Spain (PI18/029). It was carried out in accordance with the Helsinki Declaration. All of the subjects completed an informed consent form, and their data was anonymized.

## Author Contributions

SN-G and BO led the design, developed the study, had the original idea, and wrote the first draft of the manuscript. RB, SN-G, BV-S, MP, and BO coordinated the investigation. AA-L, SN-G, and BO undertook the data curation and formal analysis. The rest of the signing authors have read the manuscript critically, made contributions, and approved the final version. The corresponding author attested that all listed authors meet authorship criteria and that no others meeting the criteria have been omitted.

## Conflict of Interest

The authors declare that the research was conducted in the absence of any commercial or financial relationships that could be construed as a potential conflict of interest.

## References

[B1] Abravanel B. T. Sinha R. (2015). Emotion dysregulation mediates the relationship between lifetime cumulative adversity and depressive symptomatology. *J. Psychiatr. Res.* 61 89–96. 10.1016/j.jpsychires.2014.11.012 25528603 PMC4308421

[B2] Andrews B. (1995). Bodily shame as a mediator between abusive experiences and depression. *J. Abnorm. Psychol.* 104 277–285. 10.1037//0021-843x.104.2.277 7790630

[B3] Aragonès E. Piñol J. L. Labad A. Masdéu R. M. Pino M. Cervera J. (2004). Prevalence and determinants of depressive disorders in primary care practice in Spain. *Int. J. Psychiatry Med.* 34 21–35. 10.2190/C25N-W4NY-BN8W-TXN2 15242139

[B4] Asensio-Martínez A. Oliván-Blázquez B. Montero-Marín J. Masluk B. Fueyo-Díaz R. Gascón-Santos S. (2020). Relation of the psychological constructs of resilience, mindfulness, and self-compassion on the perception of physical and mental health. *Psychol. Res. Behav. Manag.* 12 1155–1166. 10.2147/PRBM.S225169 PMC6939394 31920412

[B5] Bellón J. A. Moreno-Küstner B. Torres-González F. Montón-Franco C. GildeGómez-Barragán M. J. Sánchez-Celaya M. (2008). Predicting the onset and persistence of episodes of depression in primary health care. The predictD-Spain study: methodology. *BMC Public Health* 8:256. 10.1186/1471-2458-8-256 18657275 PMC2527330

[B6] Bernet C. Z. Stein M. B. (1999). Relationship of childhood maltreatment to the onset and course of major depression in adulthood. *Depress. Anxiety* 9 169–174. 10431682

[B7] Bernstein D. P. Fink L. (1998). *Childhood Trauma Questionnaire: A Retrospective Self-Report Manual.* San Antonio, TX: The Psychological Corporation.

[B8] Bifulco A. Moran P. M. Baines R. Bunn A. Stanford K. (2002). Exploring psychological abuse in childhood: II. Association with other abuse and adult clinical depression. *Bull. Menning. Clin.* 66 241–258. 10.1521/bumc.66.3.241.23366 12448629

[B9] Bobes J. Bulbena A. Luque A. Dal-Ré R. Ballesteros J. Ibarra N. (2003). [A comparative psychometric study of the Spanish versions with 6, 17, and 21 items of the hamilton depression rating scale]. *Med. Clin.* 120 693–700. 10.1016/s0025-7753(03)73814-7 12781095

[B10] Bockting C. L. Hollon S. D. Jarrett R. B. Kuyken W. Dobson K. (2015). A lifetime approach to major depressive disorder: the contributions of psychological interventions in preventing relapse and recurrence. *Clin. Psychol. Rev* 41 16–26. 10.1016/j.cpr.2015.02.003 25754289

[B11] Buckman J. E. J. Underwood A. Clarke K. Saunders R. Hollon S. D. Fearon P. (2018). Risk factors for relapse and recurrence of depression in adults and how they operate: a four-phase systematic review and meta-synthesis. *Clin. Psychol. Rev.* 64 13–38. 10.1016/j.cpr.2018.07.005 30075313 PMC6237833

[B12] Bukh J. D. Andersen P. K. Kessing L. V. (2016a). Personality and the long-term outcome of first-episode depression. *J. Clin. Psychiatry* 77 e704–e710. 10.4088/JCP.15m09823 27232945

[B13] Bukh J. D. Andersen P. K. Kessing L. V. (2016b). Rates and predictors of remission, recurrence and conversion to bipolar disorder after the first lifetime episode of depression–a prospective 5-year follow-up study. *Psychol. Med.* 46 1151–1161. 10.1017/S0033291715002676 26743873

[B14] Burcusa S. L. Iacono W. G. (2007). Risk for recurrence in depression. *Clin. Psychol. Rev.* 27 959–985. 17448579 10.1016/j.cpr.2007.02.005 PMC2169519

[B15] Cassano P. Fava M. (2002). Depression and public health: an overview. *J. Psychos. Res.* 53 849–857. 10.1016/S0022-3999(02)00304-5 12377293

[B16] Chao S. F. (2014). Functional disability and depressive symptoms: longitudinal effects of activity restriction, perceived stress, and social support. *Aging Ment. Health* 18 767–776. 10.1080/13607863.2013.878308 24479794

[B17] Colman I. Naicker K. Zeng Y. Ataullahjan A. Senthilselvan A. Patten S. B. (2011). Predictors of long-term prognosis of depression. *CMAJ* 183 1969–1976. 10.1503/cmaj.110676 22025655 PMC3225418

[B18] Conejo-Cerón S. Moreno-Peral P. Rodríguez-Morejón A. Motrico E. Navas-Campaña D. Rigabert A. (2017). Effectiveness of psychological and educational interventions to prevent depression in primary care: a systematic review and meta-analysis. *Ann. Fam. Med.* 15 262–271. 10.1370/afm.2031 28483893 PMC5422089

[B19] Conradi H. J. de Jonge P. Ormel J. (2008). Prediction of the three-year course of recurrent depression in primary care patients: different risk factors for different outcomes. *J. Affect. Disord.* 105 267–271. 10.1016/j.jad.2007.04.017 17574685

[B20] Daly E. J. Trivedi M. H. Wisniewski S. R. Nierenberg A. A. Gaynes B. N. Warden D. (2010). Health-related quality of life in depression: a STAR^∗^D report. *Ann. Clin. Psychiatry* 22 43–55. 20196982

[B21] Eaton W. W. Shao H. Nestadt G. Lee B. H. Bienvenu O. J. Zandi P. (2008). Population-based study of first onset and chronicity in major depressive disorder. *Arch. Gen. Psychiatry* 65:513. 10.1001/archpsyc.65.5.513 18458203 PMC2761826

[B22] Frank E. Prien R. Jarrett R. Keller M. Kupfer D. Lavori P. (1991). Conceptualization and rationale for consensus definitions of terms in major depressive disorder. Remission, recovery, relapse, and recurrence. *Arch. Gen. Psychiatry* 48 851–855. 1929776 10.1001/archpsyc.1991.01810330075011

[B23] Gabilondo A. Rojas-Farreras S. Rodráguez A. Ferníndez A. Pinto-Meza A. Vilagut G. (2011). Use of primary and specialized mental health care for a major depressive episode in Spain by ESEMeD respondents. *Psychiatr. Serv.* 62 152–161. 10.1176/ps.62.2.pss6202_0152 21285093

[B24] Gabilondo A. Rojas-Farreras S. Vilagut G. Haro J. M. Fernández A. Pinto-Meza A. (2010). Epidemiology of major depressive episode in a southern European country: results from the ESEMeD-Spain project. *J. Affect. Disord.* 120 76–85. 10.1016/j.jad.2009.04.016 19428121 PMC3756284

[B25] Gao K. Su M. Sweet J. Calabrese J. R. (2019). Correlation between depression/anxiety symptom severity and quality of life in patients with major depressive disorder or bipolar disorder. *J. Affect. Disord.* 244 9–15. 10.1016/j.jad.2018.09.063 30292023

[B26] Gili M. Garcia-Toro M. Vives M. Armengol S. Garcia-Campayo J. Soriano J. B. (2011). Medical comorbidity in recurrent versus first-episode depressive patients. *Acta Psychiatr. Scand.* 123 220–227. 10.1111/j.1600-0447.2010.01646.x 21118188

[B27] Gili M. Roca M. Basu S. McKee M. Stuckler D. (2013). The mental health risks of economic crisis in Spain: evidence from primary care centres, 2006 and 2010. *Eur. J. Public Health* 23 103–108. 10.1093/eurpub/cks035 23132877

[B28] Gili M. Vicens C. Roca M. Andersen P. McMillan D. (2015). Interventions for preventing relapse or recurrence of depression in primary health care settings: a systematic review. *Prevent. Med.* 76 S16–S21. 10.1016/j.ypmed.2014.07.035 25192769

[B29] Gilman S. E. Kawachi I. Fitzmaurice G. M. Buka L. (2003). Socio-economic status, family disruption and residential stability in childhood: relation to onset, recurrence and remission of major depression. *Psychol. Med.* 33 1341–1355. 10.1017/s0033291703008377 14672243

[B30] González De Rivera J. L. Morera A. (1983). La valoración de sucesos vitales: adaptación española de la escala de Holmes y Rahe. *Psiquis* 4 5–9.

[B31] Gopinath S. Katon W. J. Russo J. E. Ludman E. J. (2007). Clinical factors associated with relapse in primary care patients with chronic or recurrent depression. *J. Affect. Disord.* 101 57–63. 10.1016/j.jad.2006.10.023 17156852

[B32] Grilo C. M. Stout R. L. Markowitz J. C. Sanislow C. A. Ansell E. B. Skodol A. E. (2010). Personality disorders predict relapse after remission from an episode of major depressive disorder: a 6-year prospective study. *J. Clin. Psychiatry* 71 1629–1635. 10.4088/JCP.08m04200gre 20584514 PMC4615714

[B33] Gum A. M. King-Kallimanis B. Kohn R. (2009). Prevalence of mood, anxiety, and substance-abuse disorders for older americans in the national comorbidity survey-replication. *Am. J. Geriatr. Psychiatry* 17 769–781. 10.1097/JGP.0b013e3181ad4f5a 19700949

[B34] Hamilton M. (1967). Development of a rating scale for primary depressive illness. *Br. J. Soc. Clin. Psychol.* 6 278–296. 6080235 10.1111/j.2044-8260.1967.tb00530.x

[B35] Hammen C. (1991). Generation of stress in the course of unipolar depression. *J. Abnorm. Psychol.* 100 555–561. 10.1037//0021-843x.100.4.555 1757669

[B36] Hardeveld F. Spijker J. De Graaf R. Hendriks S. M. Licht C. M. M. Nolen W. A. (2013a). Recurrence of major depressive disorder across different treatment settings: results from the NESDA study. *J. Affect. Disord.* 147 225–231. 10.1016/j.jad.2012.11.008 23218899

[B37] Hardeveld F. Spijker J. De Graaf R. Nolen W. A. Beekman A. T. F. (2009). Prevalence and predictors of recurrence of major depressive disorder in the adult population. *Acta Psychiatr. Scand.* 122 184–191. 10.1111/j.1600-0447.2009.01519.x 20003092

[B38] Hardeveld F. Spijker J. De Graaf R. Nolen W. A. Beekman A. T. F. (2013b). Recurrence of major depressive disorder and its predictors in the general population: results from The Netherlands Mental Health Survey and Incidence Study (NEMESIS). *Psychol. Med.* 43 39–48. 10.1017/S0033291712002395 23111147

[B39] Harkness K. L. Bruce A. E. Lumley M. N. (2006). The role of childhood abuse and neglect in the sensitization to stressful life events in adolescent depression. *J. Abnorm. Psychol.* 115 730–741. 10.1037/0021-843X.115.4.730 17100530

[B40] Haro J. M. Palacín C. Vilagut G. Martínez M. Bernal M. Luque I. (2006). [Prevalence of mental disorders and associated factors: results from the ESEMeD-Spain study]. *Med. Clin.* 126 445–451. 10.1157/13086324 16620730

[B41] Hernandez A. Gallardo-Pujol D. Pereda N. Arntz A. Bernstein D. P. Gaviria A. M. (2013). Initial validation of the Spanish childhood trauma questionnaire-short form: factor structure, reliability and association with parenting. *J. Interpers. Violence* 28 1498–1518. 10.1177/0886260512468240 23266990

[B42] Holmes T. H. Rahe R. H. (1967). The social readjustment rating scale. *J. Psychosom. Res.* 11 213–218. 10.1016/0022-3999(67)90010-4 6059863

[B43] Ibrahim M. Thearle M. S. Krakoff J. Gluck M. E. (2016). Perceived stress and anhedonia predict short-and long-term weight change, respectively, in healthy adults. *Eat. Behav.* 21 214–219. 10.1016/j.eatbeh.2016.03.009 27002703 PMC4851568

[B44] Karsten J. Hartman C. A. Smit J. H. Zitman F. G. Beekman A. T. F. Cuijpers P. (2011). Psychiatric history and subthreshold symptoms as predictors of the occurrence of depressive or anxiety disorder within 2 years. *Br J Psychiatry* 198 206–212. 10.1192/bjp.bp.110.080572 21357879

[B45] Katon W. J. (2003). Clinical and health services relationships between major depression, depressive symptoms, and general medical illness. *Biol. Psychiatry* 54 216–226. 10.1016/S0006-3223(03)00273-7 12893098

[B46] Kessler R. C. Berglund P. Demler O. Jin R. Koretz D. Merikangas K. R. (2003). The epidemiology of major depressive disorder. *JAMA* 289:3095. 10.1001/jama.289.23.3095 12813115

[B47] Kessler R. C. Birnbaum H. G. Shahly V. Bromet E. Hwang I. McLaughlin K. A. (2010). Age differences in the prevalence and co-morbidity of DSM-IV major depressive episodes: results from the WHO World Mental Health Survey Initiative. *Depress. Anxiety* 27 351–364. 10.1002/da.20634 20037917 PMC3139270

[B48] Kessler R. C. Magee W. J. (1994). Childhood family violence and adult recurrent depression. *J. Health Soc. Behav.* 35 13–27. 8014427

[B49] Kessler R. C. Ormel J. Petukhova M. McLaughlin K. A. Green J. G. Russo L. J. (2011). Development of lifetime comorbidity in the World Health Organization world mental health surveys. *Arch. Gen. Psychiatry* 68 90–100. 10.1001/archgenpsychiatry.2010.180 21199968 PMC3057480

[B50] Kim S. E. Kim H. N. Cho J. Kwon M. J. Chang Y. Ryu S. (2016). Direct and indirect effects of five factor personality and gender on depressive symptoms mediated by perceived stress. *PLoS One* 11:e0154140. 10.1371/journal.pone.0154140 27120051 PMC4847785

[B51] Kiosses D. N. Alexopoulos G. S. (2013). The prognostic significance of subsyndromal symptoms emerging after remission of late-life depression. *Psychol. Med.* 43 341–350. 10.1017/S0033291712000967 22607988 PMC3571621

[B52] Klein D. N. Glenn C. R. Kosty D. B. Seeley J. R. Rohde P. Lewinsohn P. M. (2013). Predictors of first lifetime onset of major depressive disorder in young adulthood. *J. Abnorm. Psychol.* 122 1–6. 10.1037/a0029567 22889243 PMC3570686

[B53] Knight M. J. Lyrtzis E. Baune B. T. (2020). The association of cognitive deficits with mental and physical quality of life in major depressive disorder. *Compr. Psychiatry* 97:152147. 10.1016/j.comppsych.2019.152147 31838296

[B54] Kool S. Dekker J. De Jonghe F. De Jong P. Schouws S. (2000). Personality disorders and social functioning in depressed patients. *Soc. Behav. Pers.* 28 163–176. 10.2224/sbp.2000.28.2.163

[B55] Lin E. H. B. Katon W. J. VonKorff M. Russo J. E. Simon G. E. Bush T. M. (1998). Relapse of depression in primary care: rate and clinical predictors. *Arch. Fam. Med.* 7 443–449. 10.1001/archfami.7.5.443 9755737

[B56] Mayer S. E. Lopez-Duran N. L. Sen S. Abelson J. L. (2018). Chronic stress, hair cortisol and depression: a prospective and longitudinal study of medical internship. *Psychoneuroendocrinology* 92 57–65. 10.1016/j.psyneuen.2018.03.020 29627713 PMC5924646

[B57] McClintock S. M. Husain M. M. Wisniewski S. R. Nierenberg A. A. Stewart J. W. Trivedi M. H. (2011). Residual symptoms in depressed outpatients who respond by 50% but do not remit to antidepressant medication. *J. Clin. Psychopharmacol.* 31 180–186. 10.1097/JCP.0b013e31820ebd2c 21346613 PMC3677201

[B58] Meerlo P. Havekes R. Steiger A. (2015). Chronically restricted or disrupted sleep as a causal factor in the development of depression. *Curr. Top. Behav. Neurosci.* 25 459–481. 10.1007/7854_2015_367 25646723

[B59] Melchior M. Chastang J.-F. Head J. Goldberg M. Zins M. Nabi H. (2013). Socioeconomic position predicts long-term depression trajectory: a 13-year follow-up of the GAZEL cohort study. *Mol. Psychiatry* 18 112–121. 10.1038/mp.2011.116 21931321 PMC3526730

[B60] Moffett J. Mill A. C. (2014). Evaluation of the flipped classroom approach in a veterinary professional skills course. *Adv. Med. Educ. Pract.* 5 415–425. 10.2147/AMEP.S70160 25419164 PMC4235505

[B61] Monroe S. M. Harkness K. L. (2005). Life stress, the “kindling” hypothesis, and the recurrence of depression: considerations from a life stress perspective. *Psychol. Rev.* 112 417–445. 10.1037/0033-295X.112.2.417 15783292

[B62] Moussavi S. Chatterji S. Verdes E. Tandon A. Patel V. Ustun B. (2007). Depression, chronic diseases, and decrements in health: results from the World Health Surveys. *Lancet* 370 851–858. 10.1016/S0140-6736(07)61415-9 17826170

[B63] Mueller T. I. Leon A. C. Keller M. B. Solomon D. A. Endicott J. Coryell W. (1999). Recurrence after recovery from major depressive disorder during 15 years of observational follow-up. *Am. J. Psychiatry* 156 1000–1006. 10.1176/ajp.156.7.1000 10401442

[B64] Nanni V. Uher R. Danese A. (2012). Childhood maltreatment predicts unfavorable course of illness and treatment outcome in depression: a meta-analysis. *Am. J. Psychiatry* 169 141–151. 10.1176/appi.ajp.2011.11020335 22420036

[B65] Nierenberg A. A. Husain M. M. Trivedi M. H. Fava M. Warden D. Wisniewski S. R. (2010). Residual symptoms after remission of major depressive disorder with citalopram and risk of relapse: a STAR^∗^D report. *Psychol. Med.* 40 41–50. 10.1017/S0033291709006011 19460188 PMC5886713

[B66] Noteboom A. Beekman A. T. F. Vogelzangs N. Penninx B. W. J. H. (2016). Personality and social support as predictors of first and recurrent episodes of depression. *J. Affect. Disord.* 190 156–161. 10.1016/j.jad.2015.09.020 26519635

[B67] O’Neil A. Jacka F. N. Quirk S. E. Cocker F. Taylor C. B. Oldenburg B. (2015). A shared framework for the common mental disorders and non-communicable disease: key considerations for disease prevention and control. *BMC Psychiatry* 15:15. 10.1186/s12888-015-0394-0 25652365 PMC4342822

[B68] Post R. M. (1992). Transduction of psychosocial stress into the neurobiology of recurrent affective disorder. *Am. J. Psychiatry* 149 999–1010. 10.1176/ajp.149.8.999 1353322

[B69] Purriños M. J. (2013). Escala de hamilton - hamilton depresion rating scale (HDRS). *Serv. Epidemiol.* 2 1–4.

[B70] Ramos-Brieva J. A. Cordero-Villafafila A. (1988). A new validation of the hamilton rating scale for depression. *J. Psychiatr. Res.* 22 21–28. 10.1016/0022-3956(88)90024-6 3397906

[B71] Read J. R. Sharpe L. Modini M. Dear B. F. (2017). Multimorbidity and depression: a systematic review and meta-analysis. *J. Affect. Disord.* 221 36–46. 10.1016/j.jad.2017.06.009 28628766

[B72] Revilla L. Luna J. Bailón E. Medina I. (2005). Validation of the MOS questionnaire of social support in primary care. *Med. Fam.* 10 10–18.

[B73] Richards D. (2011). Prevalence and clinical course of depression: a review. *Clin. Psychol. Rev.* 31 1117–1125. 21820991 10.1016/j.cpr.2011.07.004

[B74] Roca M. Armengol S. García-García M. Rodriguez-Bayón A. Ballesta I. Serrano M. J. (2011). Clinical differences between first and recurrent episodes in depressive patients. *Compr. Psychiatry* 52 26–32. 10.1016/j.comppsych.2010.04.011 21220062

[B75] Roca M. Gili M. Garcia-Garcia M. Salva J. Vives M. Garcia Campayo J. (2009). Prevalence and comorbidity of common mental disorders in primary care. *J. Affect. Disord.* 119 52–58. 10.1016/j.jad.2009.03.014 19361865

[B76] Sarubin N. Goerigk S. Padberg F. Jobst A. Erfurt L. Schumann C. (2018). Self-esteem fully mediates positive life events and depressive symptoms in a sample of 173 patients with affective disorders. *Psychol. Psychother.* 93 21–35. 10.1111/papt.12205 30488539

[B77] Sherbourne C. D. Stewart A. L. (1991). The MOS social support survey. *Soc. Sci. Med.* 32 705–714. 10.1016/0277-9536(91)90150-b 2035047

[B78] Simon G. E. (2000). Long-term prognosis of depression in primary care. *Bull. World Health Organ.* 78 439–445. 10885162 PMC2560746

[B79] Solomon D. A. (2000). Multiple recurrences of major depressive disorder. *Am. J. Psychiatry* 157 229–233. 10.1176/appi.ajp.157.2.229 10671391

[B80] Solomon D. A. Keller M. B. Leon A. C. Mueller T. I. Lavori P. W. Shea M. T. (2000). Multiple recurrences of major depressive disorder. *Am. J. Psychiatry* 157 229–233. 10.1176/appi.ajp.157.2.229 10671391

[B81] Spanish Ministry of Health Social Services Equality (2011). *National Health Survey.* Available online at: https://www.mscbs.gob.es/en/estadEstudios/estadisticas/encuestaNacional/encuesta2011.htm (accessed August 20, 2017).

[B82] Spijker J. De Graaf R. Bijl R. V. Beekman A. T. F. Ormel J. Nolen W. A. (2002). Duration of major depressive episodes in the general population: results from the netherlands mental health survey and incidence Study (NEMESIS). *Br. J. Psychiatry* 181 208–213. 10.1192/bjp.181.3.208 12204924

[B83] Springer K. W. Sheridan J. Kuo D. Carnes M. (2003). The long-term health outcomes of childhood abuse. An overview and a call to action. *J. Gen. Intern. Med.* 18 864–870. 10.1046/j.1525-1497.2003.20918.x 14521650 PMC1494926

[B84] Stegenga B. T. Geerlings M. I. Torres-González F. Xavier M. Švab I. Penninx B. W. (2013). Risk factors for onset of multiple or long major depressive episodes versus single and short episodes. *Soc. Psychiatry Psychiatr. Epidemiol.* 48 1067–1075. 10.1007/s00127-012-0626-2 23179095

[B85] Tang A. L. Thomas S. J. Larkin T. (2019). Cortisol, oxytocin, and quality of life in major depressive disorder. *Qual. Life Res.* 28 2919–2928. 10.1007/s11136-019-02236-3 31227958

[B86] ten Doesschate M. C. Bockting C. L. H. Koeter M. W. J. Schene A. H. (2010). Prediction of recurrence in recurrent depression. *J. Clin. Psychiatry* 71 984–991. 10.4088/JCP.08m04858blu 20797379

[B87] Thimm J. C. Wang C. E. A. Waterloo K. Eisemann M. Halvorsen M. (2018). Coping, thought suppression, and perceived stress in currently depressed, previously depressed, and never depressed individuals. *Clin. Psychol. Psychother.* 25 401–407. 10.1002/cpp.2173 29314412

[B88] Trainor K. Mallett J. Rushe T. (2013). Age related differences in mental health scale scores and depression diagnosis: adult responses to the CIDI-SF and MHI-5. *J. Affect. Disord.* 151 639–645. 10.1016/j.jad.2013.07.011 23993442

[B89] van Loo H. M. Aggen S. H. Gardner C. O. Kendler K. S. (2015). Multiple risk factors predict recurrence of major depressive disorder in women. *J. Affect. Disord.* 180 52–61. 10.1016/j.jad.2015.03.045 25881281 PMC4504430

[B90] Vuorilehto M. Melartin T. Isometsä E. (2005). Depressive disorders in primary care: recurrent, chronic, and co-morbid. *Psychol. Med.* 35 673–682. 10.1017/S0033291704003770 15918344

[B91] Vuorilehto M. S. Melartin T. K. Isometsä E. T. (2009). Course and outcome of depressive disorders in primary care: a prospective 18-month study. *Psychol. Med.* 39 1697–1707. 10.1017/S0033291709005182 19250580

[B92] Yiend J. Paykel E. Merritt R. Lester K. Doll H. Burns T. (2009). Long term outcome of primary care depression. *J. Affect. Disord.* 118 79–86. 10.1016/j.jad.2009.01.026 19246103

[B93] Zhai L. Zhang H. Zhang D. (2015). Sleep duration and depression among adults: a meta-analysis of prospective studies. *Depress. Anxiety* 32 664–670. 10.1002/da.22386 26047492

[B94] Zhang Y. Chen Y. Ma L. (2018). Depression and cardiovascular disease in elderly: current understanding. *J. Clin. Neurosci.* 47 1–5. 10.1016/j.jocn.2017.09.022 29066229

