# Corrigendum: Factors Associated With Depressive Episode Recurrences in Primary Care: A Retrospective, Descriptive Study

**DOI:** 10.3389/fpsyg.2020.622141

**Published:** 2020-11-25

**Authors:** Shysset Nuggerud-Galeas, Bárbara Oliván Blázquez, María Cruz Perez Yus, Begoña Valle-Salazar, Alejandra Aguilar-Latorre, Rosa Magallón Botaya

**Affiliations:** ^1^Institute for Health Research Aragón (IIS Aragón), Zaragoza, Spain; ^2^Department of Medicine, Psychiatry and Dermatology, University of Zaragoza, Zaragoza, Spain; ^3^Department of Psychology and Sociology, University of Zaragoza, Zaragoza, Spain; ^4^Primary Health Care, Aragón Health Service, Zaragoza, Spain

**Keywords:** depression, primary care, recurrence, influencing factors, retrospective

In the original article, we neglected to include the funder Feder Funds “Another way to make Europe.” The corrected funding statement appears below.

The authors apologize for this error and state that this does not change the scientific conclusions of the article in any way. The original article has been updated.

## Funding

This work was supported by Carlos III Health Institute (ISCIII) grant number PI18/01336.

The authors declare that this study received funding from Carlos III Health Institute (ISCIII)—Feder Funds “Another way to make Europe.” The funder was not involved in the study design, collection, analysis, interpretation of data, the writing of this article or the decision to submit it for publication.

